# Infantile Hemangioma of the Upper Lip: Report of a Rare Case With a Brief Review of Literature

**DOI:** 10.7759/cureus.42556

**Published:** 2023-07-27

**Authors:** Shamimul Hasan, Ateeba Khan, Abhishek Banerjee, Karthikeyan Ramalingam

**Affiliations:** 1 Oral Medicine and Radiology, Faculty of Dentistry, Jamia Millia Islamia, New Delhi, IND; 2 Oral and Maxillofacial Pathology, Awadh Dental College and Hospital, Jamshedpur, IND; 3 Oral Pathology and Microbiology, Saveetha Dental College and Hospitals, Saveetha Institute of Medical and Technical Sciences, Saveetha University, Chennai, IND

**Keywords:** oral pathology, vascular lesion, lip, venous malformations, oral cavity, hemangioma, hamartoma

## Abstract

Hamartomas are tumor-like abnormalities typified by the presence of cellular proliferation indigenous to the native site. However, hamartomas maintain growth cessation without the potential for further growth or malignant transformation. Hamartomas are commonly seen in the lungs, kidney, liver, and spleen and rarely occur in the orofacial region. Various hamartomatous oral lesions include hemangiomas, lymphangiomas, tori, exostosis, dens invaginatus, dens evaginatus, odontomas, nevi, and cherubism. Infantile hemangiomas are benign vascular tumors that emerge soon after birth and experience rapid growth within the first year. Oral hemangiomas affect up to 6.4% of infants and are more common on the ventral surface of the tongue, as opposed to oral vascular malformations, which are more prevalent on the lips. It also has a 3:1 female-to-male predominance. Afterward, their growth usually stabilizes and enters a prolonged, incomplete involution phase. Uncomplicated hemangiomas generally exhibit spontaneous resolution, whereas few can leave behind scars and telangiectasias on the external surface of the skin on which it occurs. Thus, lesions located in anatomically sensitive regions necessitate vigilant surveillance and treatment. This paper deals with an asymptomatic swelling of the upper lip in a four-year-old female child but with problems in aesthetics, speech, and feeding. A thorough history, clinical examination, positive diascopy, ultrasonography, and histopathology confirmed the diagnosis of infantile hemangioma.

## Introduction

The term “hamartoma” is derived from the Greek words "hamartia" (defect, error) and "oma" (tumor-like growth) [1]. Hamartoma is defined as a non-neoplastic, unifocal/multifocal, developmental abnormality, encompassing a blend of cytologically normal mature tissues and cells that are native to the anatomic site, and exhibits a disordered architectural pattern with a preponderance of one of its components. The lung, spleen, pancreas, liver, and kidney are the frequent sites of occurrence. However, the entity rarely occurs in the head and neck region [2].

The characteristic attributes of hamartomas are that they are benign and non-encapsulated, exhibit a self-limiting growth potential, cease to grow at some point of course, and do not infiltrate into adjacent tissues [3]. The pathogenesis of hamartomas is still obscure. They may emanate from any one of the embryonic derivates, most frequently the mesoderm. This is almost never in the case of neoplasm, where the neoplastic cells are clonally derived [4].

Hemangiomas conventionally represent a range of developmental vascular disorders and are assigned terms, such as hamartoma, true benign neoplasm/tumors, or malformations. It accounts for the most common hamartomas in the orofacial region among infants and children. They are broadly classified into capillary-port wine stain, cavernous-salmon patch, and miscellaneous forms, such as verrucous, venous, and arteriovenous hemangiomas. They usually manifest in the first month of life and can show rapid proliferation and slow involution. Complications include ulceration, hemorrhage, dysphagia, airway compromise, and speech impairment based on its location [5,6,7]. 

The International Society for the Study of Vascular Anomalies acknowledged this classification and introduced a universally accepted revised nomenclature in 2014 [8,9,10]. This standardized classification has not only facilitated a precise evaluation of the true incidence of infantile hemangioma (IH) but also has rendered the ideal therapeutic protocol for vascular anomalies [11].

The term hemangioma originates from Greek words "hema" (blood), "angeio" (vessel), and "oma" (tumor). Clinically, hemangiomas may occur in two forms, i.e., Infantile or congenital hemangiomas [12,13]. IHs exhibit glucose transporter protein-1 (GLUT1) positivity, in contrast to congenital hemangiomas. Besides this genetic variance, several salient characteristics can also help to differentiate the two forms [14]. IHs appear during the first two months of life and display expeditious propagation between six months and one year of age, followed by a period of slow regression [15]. On the contrary, congenital hemangiomas are present at birth and become symptomatic with an increase in size or response to infection, hormonal fluctuations, and traumatic injuries [14].

There are no standard treatment protocols for hemangiomas, as medical, interventional, and surgical options are suggested. Treatment is initiated for patients with only aesthetic or functional complaints [7].

Here, we present a rare case of hemangioma of the upper lip in a four-year-old female who presented with asymptomatic upper lip swelling associated with unaesthetic facial appearance, speech, and feeding difficulties. The patient was treated with surgical excision. 

## Case presentation

A four-year-old female child was brought by her parents to our outpatient department with a concern about a mass on her upper lip for the past 3.5 years. The patient’s parents revealed that the child was prematurely born with low birth weight to a 38-year-old female who developed pre-eclampsia. There was no facial disfigurement at birth. After four weeks of age, the lip swelling was initially noticed as a white pinhead-sized papule and exhibited a progressive increase in size for two years. However, it has remained a stabilized lesion for the last year. The parents had also noticed a color change in the lesion on crying with minimal bleeding. Medical and family history was non-significant.

**Figure 1:**
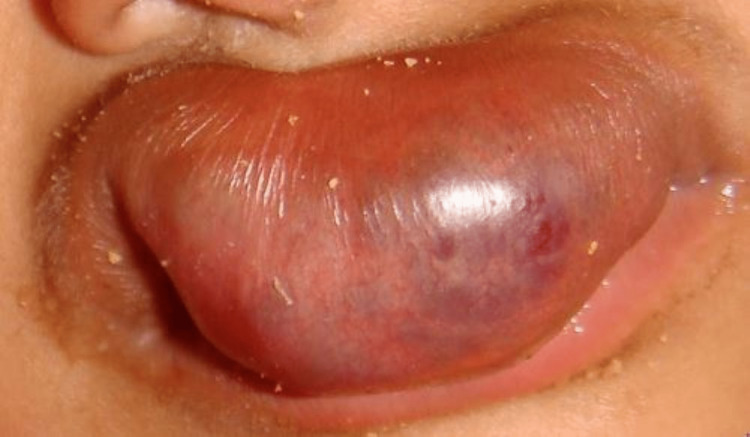
Clinical picture showing a solitary, reddish-purplish, ovoid swelling of the upper lip.

On clinical examination, a solitary ovoid-shaped, sessile, reddish-purplish swelling of the upper lip, measuring 2.5x1.5 cm in diameter, extending from the philtrum to the labial mucosa, involving the skin, mucosa, and muscle, was noticed. The swelling covered almost the entire lower lip, and only one-third of the lower lip was visible (Figure 1).

**Figure 2:**
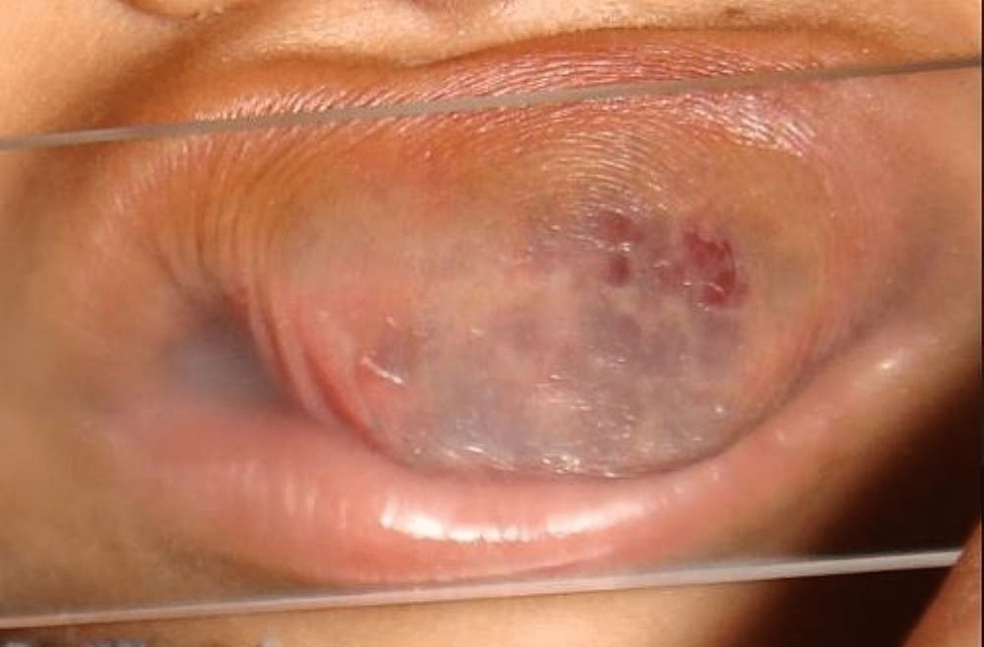
Clinical picture showing a positive diascopy test.

On closer inspection, the overlying skin was stretched and shiny with minute bleeding points and engorged vasculature. The lesion was unaesthetic and hindered feeding and speech. On palpation, the inspection findings were confirmed. The swelling was non-tender, soft to firm in consistency, compressible, and reducible and exhibited a positive diascopy test (Figure 2).

**Figure 3:**
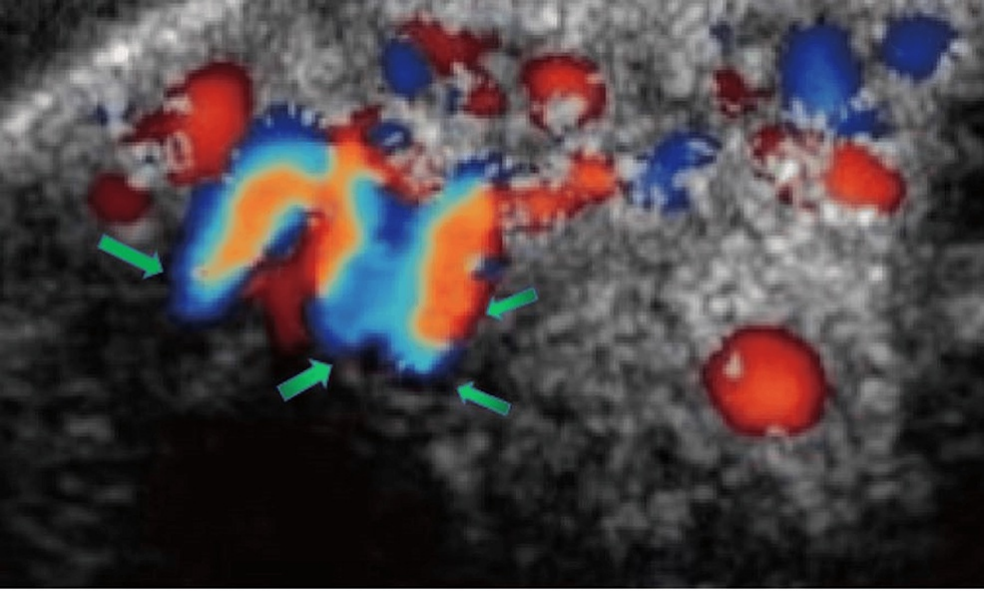
Color Doppler ultrasonography showing a hypoechoic, homogeneous, soft-tissue mass in the midline of the upper lip.

A thorough general examination ruled out the presence of any similar swelling in the body. Color-doppler ultrasonography revealed a hypoechoic, soft tissue mass shadow of 20x12 mm lying in the midline of the upper lip, with a venous type of vascularity, and no evidence of calcification or necrosis. The echotexture of the mass was homogeneous, suggesting a benign nature with no evidence of invasion to any adjacent structure (Figure 3).

**Figure 4:**
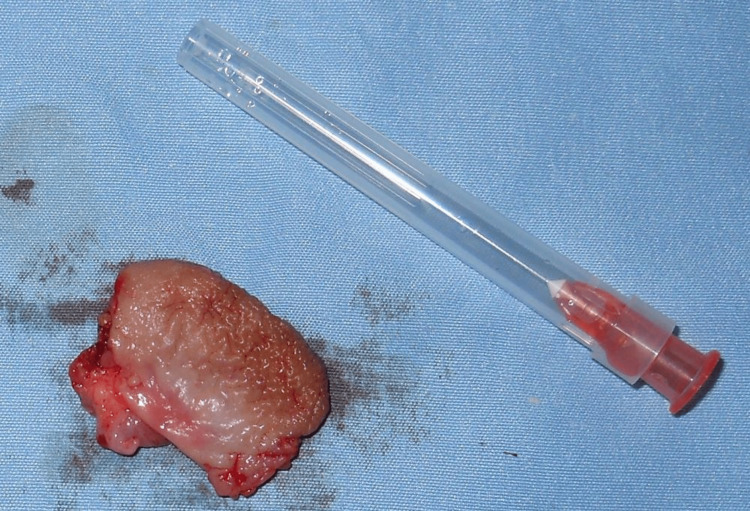
Postoperative picture showing the excised lesion specimen.

After an unremarkable hematological evaluation and obtaining written parent's consent, the patient was scheduled for surgical excision of the mass and lip reconstruction. The excised specimen, measuring about 2x1.5 cm, reddish‑brown in color, and soft in consistency, was sent for histopathological evaluation (Figure 4).

**Figure 5:**
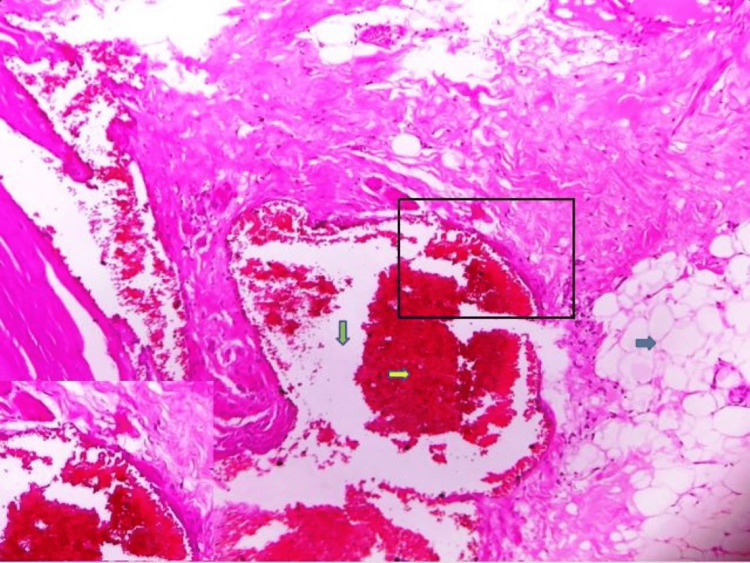
Photomicrograph showing vascular spaces filled with erythrocytes along with fibrous connective tissue and adipose tissue (hematoxylin and eosin stain, 40x).

Microscopic examination showed numerous small- to large-sized thin-walled vascular channels filled with erythrocytes and lined by endothelial cells. Mature connective tissue stroma with chronic inflammatory cell infiltration and adipose tissue are also seen (Figure 5).

Correlating the history, clinical, radiographical, and histopathological features, a final diagnosis of infantile hemangioma was made. The patient was periodically reviewed once in three months, and no recurrence was observed during the one-year follow-up.

## Discussion

The heterogeneous clinical representation of vascular deformities has resulted in a diagnostic ambiguity regarding their denotation. Mulliken and Glowacki (1982) attempted to resolve this uncertainty and were the first to elucidate a systematized terminology of vascular anomalies [7].

Based on the pathological attributes (endothelial cell turnover), they categorized the vascular deformities into two groups: (a) vasoproliferative neoplasm (hemangiomas) and (b) vascular malformations (VMs). VMs have less endothelial cell turnover (proliferate and undergo mitosis) in contrast to vasoproliferative neoplasm. Instead, VMs are structural aberrations of venous, lymphatic, capillary, and arterioles that exhibit growth in proportion to the child’s development [8]. Vascular lesions grow due to alterations in flow and pressure, vascular channel dilatation, and collateral proliferation [9].

Despite the clinical similarities, hemangiomas and VMs have few distinct histopathologic and histochemical features, which facilitate the establishment of a definite diagnosis [12] (Table 1).

**Table 1:** Difference between hemangioma and vascular malformations. Source: Olsen et al. [12]

Hemangiomas	Vascular malformation
A hemangioma may or may not be present at birth.	A vascular malformation is always present at birth.
They are true benign neoplasms of endothelial cells.	They are localized defects of vascular architecture that result in the formation of abnormal torturous and enlarged vascular channels.
Females are more commonly affected compared to males (3:1).	Vascular malformations show no gender predilection.
Hemangiomas are also known as port‑wine stains, strawberry hemangiomas, and salmon patch.	Vascular malformations are also known as lymphangiomas, arteriovenous malformations, and vascular gigantism.
They grow often faster than the child’s growth.	They enlarge proportionately with the growth of the child.
Over time, they become smaller (involute) and lighter in color and may leave a scar.	They do not involute spontaneously and may become more apparent as the child grows.
Mast cells are known to play a role in neo-angiogenesis and increase during proliferating phase.	They show no increase in mast cells.

Clinically, oral hemangiomas are non-neoplastic, vascular hamartomas that appear as a non-tender, variably sized soft tissue mass with a smooth, lobulated, sessile, or pedunculated surface, with a slight female predilection [16]. Hemangiomas have a characteristic light bluish hue, with a typical “blanching” effect [16,17]. Hemangiomas are generally non-tender and can be ulcerated and hemorrhagic if traumatized. Hemangiomas exhibit a wide array of clinical manifestations, such as facial asymmetry, pulsation, scarring, spontaneous bleeding, teeth mobility, bone expansion, root resorption, paresthesia, premature primary tooth exfoliation, delayed eruption of permanent teeth, and missing teeth [18]. Our patient was a female child who presented with an asymptomatic, ovoid-shaped, sessile, reddish-purplish lip mass, with multiple bleeding points and engorged capillary plexus. 

A very prominent feature is blood oozing from the sulcus. The teeth involved may also show a pumping movement when pressure is applied to it and released (pumping tooth sign) [19]. The turkey wattle sign is an unusual pathognomic manifestation of intramasseter and intraparotid hemangiomas. It refers to the enlargement of the lesion with teeth-clenching or with dependent head positioning. The sign may be due to vascular engorgement within the lesion, which impedes venous return from the head to the superior vena cava [20]. 

Although the exact pathogenesis is still obscure, impaired vascular homeostasis due to developmental anomalies in the first trimester of pregnancy is the commonly recommended pathophysiological event [19]. The predisposing risk factors include premature low-birth-weight babies (<1000 g), increased maternal age, decreasing gestational age, white females, twins, gestational hypertension, placenta previa, multiple gestation pregnancy, chronic villus sampling, and antenatal vaginal bleeding [20]. Our patient was a four-year-old, premature, low-birth-weight child born to a 38-year-old female who developed pre-eclampsia.

Port-wine stains (PWS), also known as nevus flammeus, are congenital, cutaneous capillary malformations over the forehead and nose, overlying the distribution of trigeminal nerve branches (V1, V2, and V3), and persist throughout life. Salmon patches or nevus simplex are cutaneous venous malformations, usually on the upper eyelids, forehead (also called an angel’s kiss), and nape of the neck (also called stork bite marks). Most salmon patches disappear within one to two years of age [21]. 

These lesions comprise nests of endothelial cells that exhibit expeditious proliferation in the first year of life, followed by a slow regression. The typical history is that most hemangiomas generally appear by the third month of age and undergo a rapid proliferative phase (in which growth exceeds the child's growth), followed by slow regression [21]. In the present case, the lesion was noticed at the first month of age. However, incoherence to the published literature, the parents reported for treatment after four years only when the exceedingly large lesion resulted in an aesthetic, functional, and social disability [22].

Vascular lesions exhibit pallor and blanching on the application of a firm pressure by a flat transparent device (e.g., a glass slide). When the instrument is removed, the lesion remains pale for a few seconds; then, it slowly starts refilling again from the feeder's vessels. Perez-Lopez et al. [23] recommended that diascopy can ascertain whether the lesion color is due to blood in the vessel vasculature, which exhibits blanching (as in hemangiomas), in contrast to the extravasated blood in the tissues, which do not blanch on pressure. However, in cases where diascopy shows conflicting results, diagnostic confirmation should be made by imaging methods, such as color Doppler ultrasonography, magnetic resonance imaging (MRI), or angiography [20]. Doppler ultrasound may reveal the subtype of the vascular deformity, and MRI determines the extent of the lesion [24]. 

A positive diascopy test was revealed in our case, and color Doppler ultrasonography revealed a hypoechoic, homogeneous, soft-tissue mass in the midline of the upper lip, with a venous type of vascularity and no evidence of calcification or necrosis. Hemangiomas may exhibit a range of histopathological features, generally comprising hyperplastic endothelial cells with an augmented mitotic activity. The recently accepted classification delineates hemangiomas into three types, i.e., capillary, cavernous (large vessel), and mixed, based on the size of vascular channels [20,25].

Hemangiomas usually exhibit spontaneous regression and do not necessitate specific management protocol. Periodic evaluation and conservative therapy are the preferred treatment protocol in most cases [20]. However, exceedingly large lesions interfering with aesthetics and function accounting for 10-20% of hemangiomas require specific therapy [8,26,27,28].

**Figure 6:**
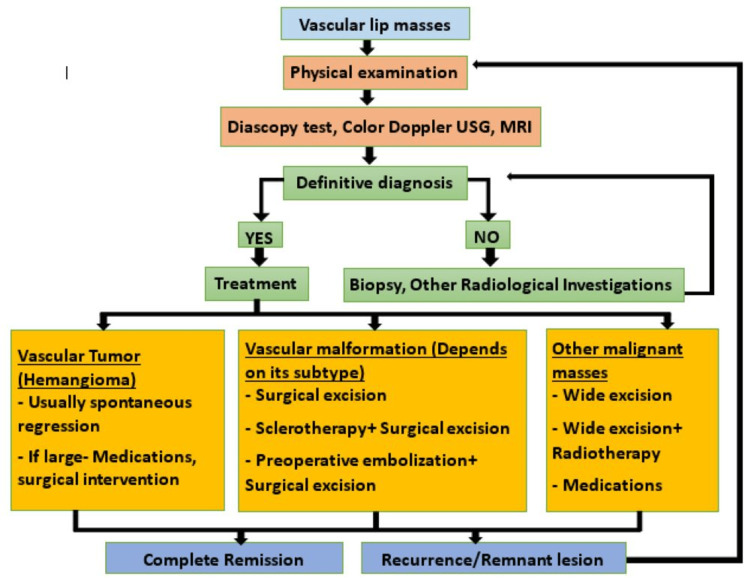
Simplified treatment algorithm for vascular lip lesions.

A wide array of therapeutic options has been recommended for hemangiomas, including non-selective beta-blockers (e.g., oral propranolol and topical timolol), intralesional corticosteroid injections, sclerotherapy (polidocanol (hydroxyl-pro ethoxy-dodecane, with 5% pure ethanol), sodium-tetradecyl-sulfate, and ethanolamine oleate), interferon-alpha, feeder vessel embolization, radiation, cryosurgery using cryoprobes (nitrous oxide gas at a temperature of -89°C at the probe tip), electrocoagulation, laser (pulsed dye laser, neodymium-doped yttrium aluminum garnet (Nd:YAG) laser, CO_2_ laser), and surgery. However, excisional biopsy surgery remains the gold standard of treatment [29,30,31,32]. Surgical excision of the mass was considered in our case due to the large-sized lesion that posed aesthetic, social, and functional disabilities. A treatment algorithm is shown in Figure 6.

The prognosis of hemangioma, in general, is excellent because it does not tend to recur or undergo malignant transformation following adequate treatment [27]. A literature search revealed that the follow-up period varied between one and eight months with a mean value of 2.7 months [20,22,24]. In our case, the patient was periodically reviewed once in three months, and no recurrence was observed during the one-year follow-up.

The differential diagnosis of diffuse upper lip swelling may include angioedema, cheilitis glandularis (CG), entities of minor salivary gland origin (mucocele, salivary duct cysts, and benign and malignant minor salivary gland tumors), infectious origin (odontogenic/non-odontogenic), mesenchymal tumors of adipose tissue (lipoma), and vascular entities (hemangioma and lymphangioma) [2]. 

Angioedema is typically characterized by non-pitting, asymmetric swelling of the face, lips, tongue, larynx, genitalia, and extremities. Angioedema typically occurs within several minutes of exposure to an identifiable allergic trigger, including food, medications, and insect stings. It rapidly subsides after administration of anti-histamines and epinephrine and does not recur without repeat insult by the allergic trigger [24].

CG is a chronic, suppurative inflammation of the lower lip, characterized by swelling of the mucous glands and associated with mucopurulent discharge through dilated ductal openings. This condition is more common in middle-aged to older males [25].

The lower lip is extremely vulnerable to factitial injury and has a propensity for minor salivary gland tissues; thus, mucocele or salivary duct cysts account for the majority of swellings/lumps in this area [2]. In the present case, the probability of the mass being a mucocele may be negated by its large size (mucoceles larger than 1.5 cm in diameter are extremely infrequent), age of the patient (mucoceles are more likely to occur in children and young adults), and chronic duration of 3.5 years. The possibility of salivary duct cysts is highly unlikely as they characteristically appear as bluish, firm nodular swellings on the floor of the mouth and rarely on the lips [2].

Benign minor salivary gland tumors (primarily pleomorphic adenomas and canalicular adenomas), although involving the upper lip, are extremely rare in infancy and childhood. Pleomorphic adenoma is usually seen in younger individuals (<40 years), and canalicular adenoma has an elderly female predilection (>60 years) [2]. The diagnosis of a malignant minor salivary gland tumor (adenoid cystic carcinoma and acinic cell carcinoma) may be ruled out based on the location, well-circumscribed mass, chronic duration, and lack of fixation [2].

Soft tissue infections of the lip (odontogenic/non-odontogenic) may be negated due to the asymptomatic and chronic duration of the lesion with normal dentition. Soft tissue lip abscess is generally ill-defined and fluctuant and exhibits inflammatory signs (pain and erythema) [2]. Lipoma is a non-neoplastic adipose tissue tumor, with an uncommon (1-4%) occurrence in the oral cavity. Lipomas characteristically manifest as a slow-growing, yellow-colored, soft, asymptomatic submucosal mass, with a typical “slip sign,” occurring mostly on the tongue and cheek region [2]. Lymphangioma, a benign hamartomatous lesion of the lymphatic system, primarily affects infants and children and involves the tongue, although few lip cases have also been documented. The lip lesions frequently exhibit an asymmetric, asymptomatic, firm, and nodular pattern [2].

Clinical implications

Lip hemangiomas pose a significant risk of complications that can adversely affect both function and appearance. These lesions may ulcerate, causing pain and bleeding, thus interfering with feeding in infants. The lips play a crucial role in defining facial features, and large hemangiomas may distort the intricate lip architecture. Surgical intervention is imperative in cases where the lip symmetry and contour are not restored despite lesion regression [33]. 

Scarring and lip contour distortion, resulting in lifelong deformity, account for the major clinically pertinent surgical complications of lip hemangiomas. Infantile lip hemangiomas often pose a surgical challenge, owing to the distinctive vermillion lip tissue, mainly the mucocutaneous junction. The reconstruction of the missing vermillion lip tissue often poses a surgical threat [34].

## Conclusions

Due to abundant vascular supply and frequent traumatic injuries, the lip accounts for a common site for a range of vascular pathologies. A meticulous detailed history, coupled with clinical, radiographic, and histologic examination, is imperative to arrive at a definite diagnosis of vascular lip lesions. Dental practitioners should be familiar with such varied clinical presentations of vascular pathologies for their prompt management.
